# Supplementary material for: Saliency models perform best for women’s and young adults' fixations
Source: Commun Psychol. 2023 Nov 17;1:34. doi: 10.1038/s44271-023-00035-8 (PMC11332104; doi:10.1038/s44271-023-00035-8)
Supplement: Supplementary file 2 — Supplementary material [file 44271_2023_35_MOESM2_ESM.pdf]

Supplementary information to: Saliency models perform best for women's and young adults' fixations

Christoph Strauch*[1], Alex J. Hoogerbrugge*[1], Gregor Baer[1], Ignace T. C. Hooge[1], Tanja C. W. Nijboer[1], Sjoerd M. Stuit[1], and Stefan Van der Stigchel[1]

[1]Experimental Psychology, Helmholtz Institute, Utrecht University, Utrecht, The Netherlands

*These authors contributed equally

Supplementary information to: Saliency models perform best for women's and young adults' fixations

All data and analysis scripts to the following analyses can be retrieved via the Open Science Framework.

Setup

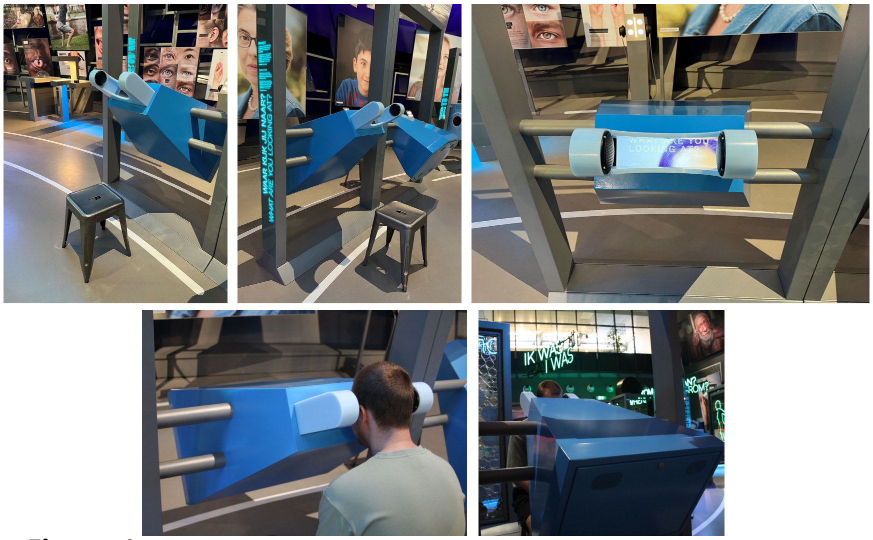

Supplementary Figure 1

Setup at the science museum. Upper left and upper center: Metal box containing the eye tracker and monitor. Upper right: view inside the metal box, 'horns' are loudspeakers used after free viewing to ask for data donation. Bottom left: starting screen. Bottom center and right: participant taking part in the study. Participants sat on a small chair.

Supplementary Figure 1 contains pictures of the setup and the context of data collection.

Further analyses across demographic groups

N = 91 participants reported non-binary gender. Due to issues with the setup, data from these participants should be interpreted with caution. Supplementary Table 1 gives absolute NSS scores per model and baseline across age bins.
Supplementary Figure 2 depicts spatial distribution maps of fixation locations per age bin.

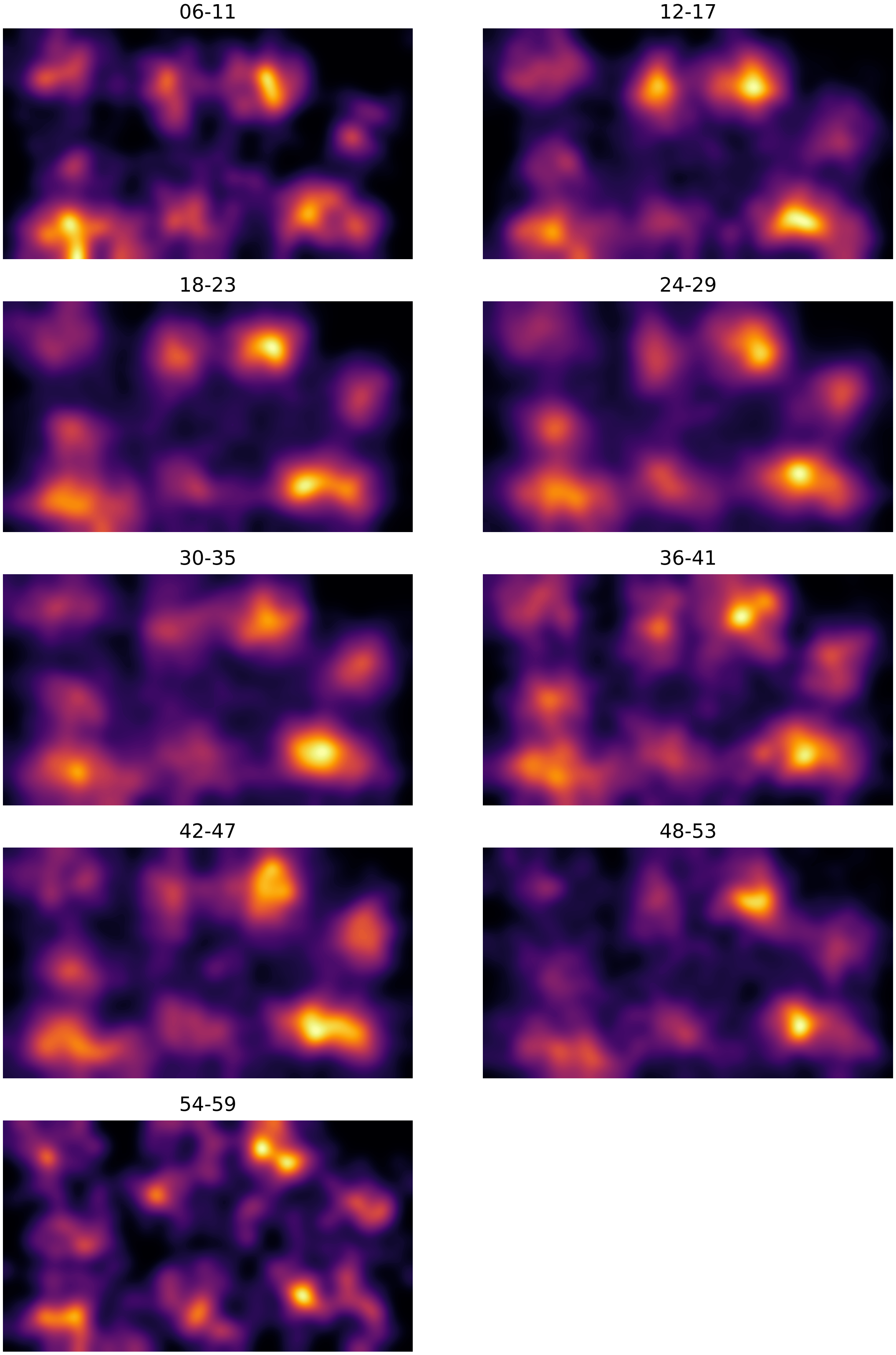

Supplementary Figure 2

Spatial distribution maps of fixation locations, separated per age bin. $n = 1{,}600$.

Supplementary Table 1

Absolute NSS values per baseline and model across age bins.

Model	M_{sample}	M_{bins}	6-11	12-17	18-23	24-29	30-35	36-41	42-47	48-53	54-59
**Baselines**											
Fixation map	0.71	0.834	1.02	0.862	0.837	0.731	0.729	0.729	0.769	0.816	1.013
Central bias	0.001	-0.005	-0.036	0.002	-0.025	0.028	0.009	-0.011	-0.034	0.034	-0.013
Single observer	0.174	0.185	0.256	0.211	0.219	0.176	0.164	0.148	0.169	0.164	0.156
Meaning map	0.382	0.367	0.338	0.343	0.409	0.414	0.382	0.365	0.361	0.361	0.33
**Models**											
RARE2012	0.194	0.182	0.163	0.147	0.185	0.226	0.197	0.188	0.185	0.216	0.129
SalGAN	0.42	0.402	0.395	0.334	0.471	0.459	0.406	0.408	0.426	0.408	0.315
DeepGazeIIE	0.405	0.392	0.396	0.392	0.44	0.435	0.401	0.361	0.378	0.407	0.32
SALICON	0.455	0.439	0.433	0.427	0.51	0.478	0.442	0.428	0.447	0.463	0.324
DVA	0.3	0.285	0.279	0.224	0.313	0.339	0.304	0.277	0.305	0.3	0.225
FES	0.052	0.043	0.051	0.014	0.039	0.083	0.064	0.042	0.029	0.059	0.007
QSS	0.338	0.328	0.365	0.294	0.363	0.364	0.338	0.304	0.332	0.337	0.259
SSR	0.265	0.252	0.257	0.206	0.297	0.278	0.283	0.257	0.246	0.28	0.163
CVS	-0.085	-0.087	-0.067	-0.076	-0.102	-0.077	-0.074	-0.091	-0.11	-0.079	-0.111
IMSIG	0.333	0.317	0.33	0.282	0.353	0.361	0.347	0.306	0.323	0.334	0.221
LDS	0.031	0.027	0.045	0.048	0.027	0.048	0.028	0.014	-0.003	0.037	-0.004
ICF	0.26	0.251	0.252	0.275	0.268	0.288	0.258	0.21	0.233	0.282	0.192
GBVS	0.166	0.156	0.134	0.171	0.151	0.207	0.157	0.142	0.13	0.2	0.113
CAS	0.161	0.154	0.177	0.171	0.158	0.18	0.161	0.135	0.134	0.187	0.086
SUN	0.167	0.165	0.183	0.147	0.161	0.183	0.176	0.141	0.147	0.206	0.144
DeepGazeI	0.334	0.319	0.322	0.311	0.363	0.365	0.328	0.294	0.311	0.346	0.232
AIM	0.26	0.259	0.283	0.264	0.269	0.276	0.259	0.21	0.237	0.297	0.234
SAM	0.258	0.255	0.254	0.337	0.288	0.27	0.215	0.225	0.236	0.288	0.182
DeepGazeII	0.4	0.382	0.373	0.341	0.42	0.446	0.396	0.37	0.383	0.393	0.317
IKN	0.202	0.188	0.174	0.164	0.194	0.247	0.202	0.18	0.171	0.223	0.135
BMS	0.186	0.175	0.216	0.182	0.202	0.201	0.192	0.159	0.155	0.194	0.074

Note: M_{sample} is the average performance across all participants analyzed here, M_{bins} is the average NSS across age bin averages per baseline/model.

Inferential statistics

Inferential statistics are given in Supplementary Table 2, including effect sizes and respective 95% confidence intervals for comparisons of NSS deviations across age bins from average across age bins (Figure 2, right). Assumed normality was not violated in any of the age bins' relative deviations to model performance, which is why one sample t-tests were conducted (Shapiro-Wilk tests, all $p > 0.073$) with the exception of the bin for 12-17 year olds, for which a Wilcoxon test was calculated ($V_{\text{Wilcoxon}} = 190.000$, $p = 0.007$, rank-biserial correlation $= 0.810$).

Supplementary Table 2

Inferential statistics across age bins for one sample t-tests on deviation in NSS across models, separated per age bin.

				95% CI	
Age bin (years)	$t(20)$	p	*Cohen's d*	Lower	Upper
6-11	1.662	0.112	0.363	-0.013	0.729
12-17	-1.453	0.162	-0.317	-0.681	0.055
18-23	4.308	< .001	0.94	0.497	1.364
24-29	10.756	< .001	2.347	1.626	3.035
30-35		0.007		0.555	0.925
36-41	-4.805	< .001	-1.048	-1.488	-0.589
42-47	-2.725	0.013	-0.595	-0.978	-0.198
48-53	9.099	< .001	1.986	1.345	2.597
54-65	-10.785	< .001	-2.354	-3.043	-1.631

Note: Confidence intervals for t-tests and Wilcoxon test (bin 30-35).

Detailed model performance across gender

Supplementary Table 3 gives model predictions in NSS. Supplementary Figure 3 depicts the spatial distribution maps of fixation locations. Note that the average difference between women and men (NSS = 0.0166) was highly similar to the difference of the central bias (NSS = 0.017). Supplementary Figure 3 depicts spatial distribution maps of fixation locations for women and men. Results for participants reporting non-binary gender are given in Supplementary Table 4 and Supplementary Figure 4 - yet caution is necessary before interpreting these results as the non-binary option was the default setting and may therefore not have been selected intentionally but could have simply been left as-is by the participant. Furthermore, there was no 'prefer not to say' option, rendering interpretation difficult.

.

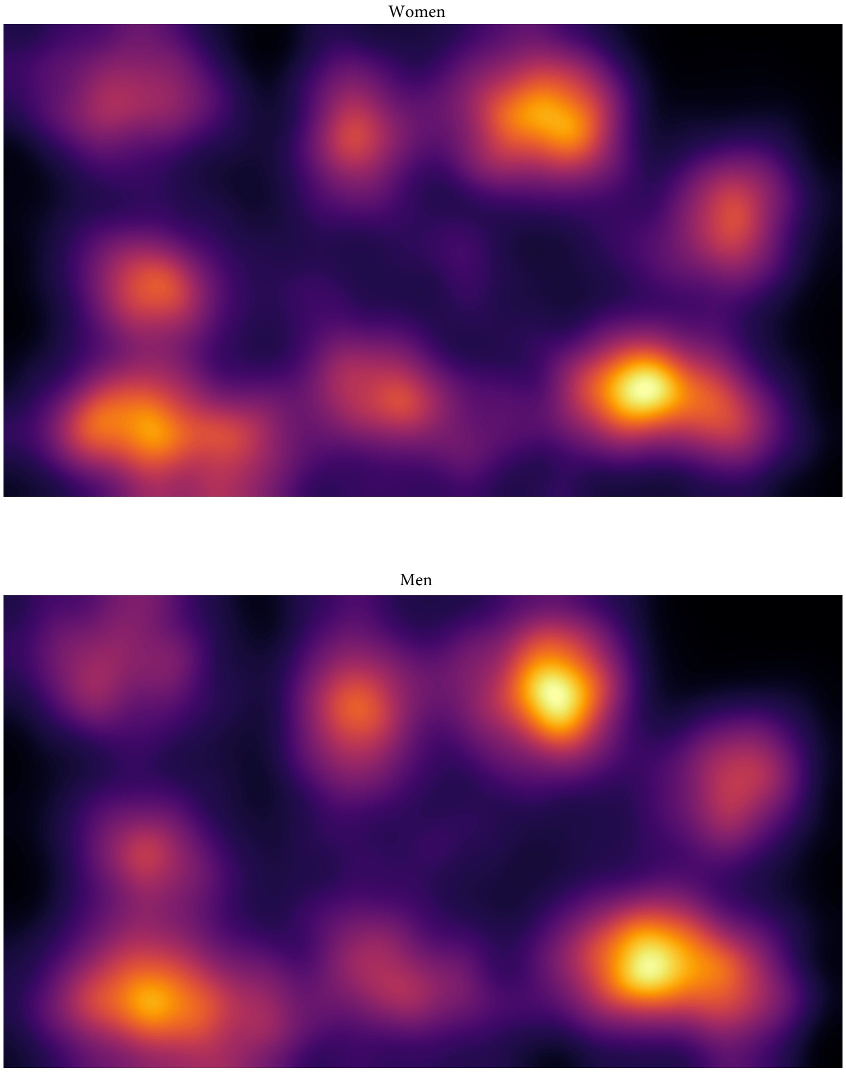

Supplementary Figure 3

Spatial distribution maps of fixation locations, separated for women (upper) and men (lower). $n = 1{,}600$.

Supplementary Table 3

Model performance for men and women.

Model	Mean	Men	Women	Difference
Fixation map	0.723	0.71	0.736	0.026
Central bias	0.002	-0.007	0.011	0.018
Single observer	0.177	0.171	0.182	0.011
Meaning map	0.384	0.376	0.392	0.016
RARE2012	0.196	0.185	0.206	0.021
SalGAN	0.422	0.425	0.419	-0.006
DeepGazeIIE	0.409	0.39	0.427	0.037
SALICON	0.458	0.445	0.471	0.026
DVA	0.303	0.289	0.317	0.028
FES	0.054	0.042	0.066	0.024
QSS	0.34	0.335	0.344	0.009
SSR	0.267	0.253	0.282	0.029
CVS	-0.086	-0.084	-0.087	-0.003
IMSIG	0.335	0.33	0.34	0.01
LDS	0.031	0.027	0.035	0.008
ICF	0.262	0.253	0.27	0.017
GBVS	0.167	0.159	0.175	0.016
CAS	0.162	0.156	0.168	0.012
SUN	0.169	0.16	0.178	0.018
DeepGazeI	0.337	0.323	0.35	0.027
AIM	0.262	0.252	0.271	0.019
SAM	0.258	0.274	0.242	-0.032
DeepGazeII	0.402	0.391	0.414	0.023
IKN	0.204	0.188	0.22	0.032
BMS	0.188	0.168	0.208	0.04

Note: NSS for the prediction of fixations for the full sample, men, and women. Relative deviations (NSS) between predictions for men and women are given in the fifth column. Positive numbers (blue) indicate better performance on women, negative numbers (red) indicate better performance on men.

Supplementary Table 4

Model performance for men, women, and participants of other gender.

	Model	Mean	Men	Women	Other
0	Fixation map	0.766	0.710	0.736	0.852
1	Central bias	0.020	-0.007	0.011	0.057
2	Single observer	0.176	0.171	0.182	0.174
3	Meaning map	0.387	0.376	0.392	0.395
4	RARE2012	0.206	0.185	0.206	0.226
5	SalGAN	0.454	0.425	0.419	0.518
6	DeepGazeIIE	0.424	0.390	0.427	0.454
7	SALICON	0.472	0.445	0.471	0.501
8	DVA	0.323	0.289	0.317	0.363
9	FES	0.071	0.042	0.066	0.106
10	QSS	0.363	0.335	0.344	0.408
11	SSR	0.283	0.253	0.282	0.316
12	CVS	-0.069	-0.084	-0.087	-0.037
13	IMSIG	0.353	0.330	0.340	0.390
14	LDS	0.048	0.027	0.035	0.081
15	ICF	0.279	0.253	0.270	0.315
16	GBVS	0.179	0.159	0.175	0.202
17	CAS	0.176	0.156	0.168	0.202
18	SUN	0.164	0.160	0.178	0.155
19	DeepGazeI	0.351	0.323	0.350	0.381
20	AIM	0.258	0.252	0.271	0.252
21	SAM	0.284	0.274	0.242	0.336
22	DeepGazeII	0.425	0.391	0.414	0.470
23	IKN	0.221	0.188	0.220	0.254
24	BMS	0.203	0.168	0.208	0.232

Note: NSS for the prediction of fixations for the full sample, men, women, and participants with other gender. Note that results for participants with other gender have to be interpreted cautiously as this represented the default option and likely contains a substantial amount of data that is not from participants identifying as non-binary.

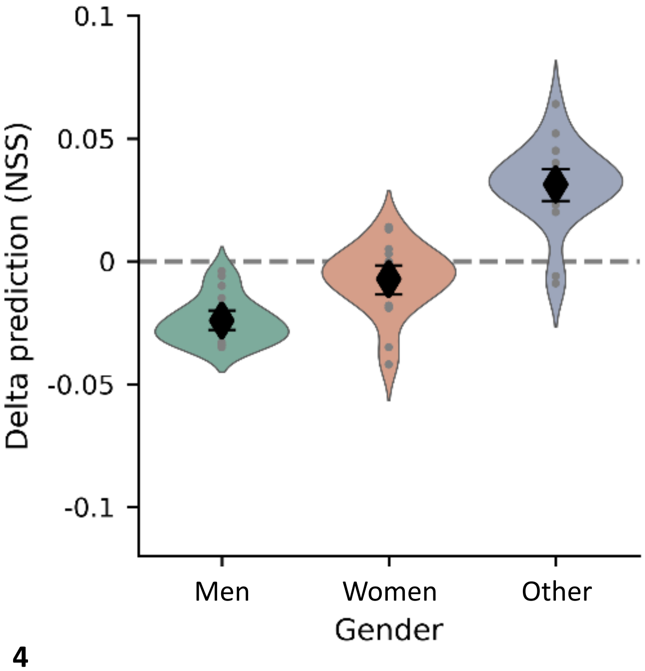

Supplementary Figure 4

Relative deviations in NSS across models for men, women, and other gender. $n = 1{,}600$ *(men, women).* $n = 91$ *(other).*

Data quality

Eye tracking data quality can be assessed by precision, accuracy, and data loss [1, 2]. In the current study, eye tracking data quality was operationalized by precision and data loss only because the experimental protocol and set up did not allow to estimate accuracy. Precision was calculated by the sample-to-sample RMS deviation (s2s-RMSd), following Hooge *et al.* [3]. This method allows for estimating the precision without removing the saccades from the gaze signal. The s2s-RMSd was determined in a window of 200 ms that was slided through the gaze signal with steps of 32 milliseconds (two samples). For each participant, the median of the s2s-RMSd over all windows was calculated. The latter value was averaged over all participants.
Median precision was 0.68° with a standard deviation of 0.28°. Data loss was $M = 0.8\%$, $SD = 2.4\%$. Women had an average data loss of 0.78% and men had an average data loss of 0.79%. Supplementary Figure 5 depicts data quality (precision in RMS and percentage data loss) across age bins and indicated gender. From the overall pattern of data quality indicators, data quality seems unlikely to have driven the main biases reported in the manuscript.

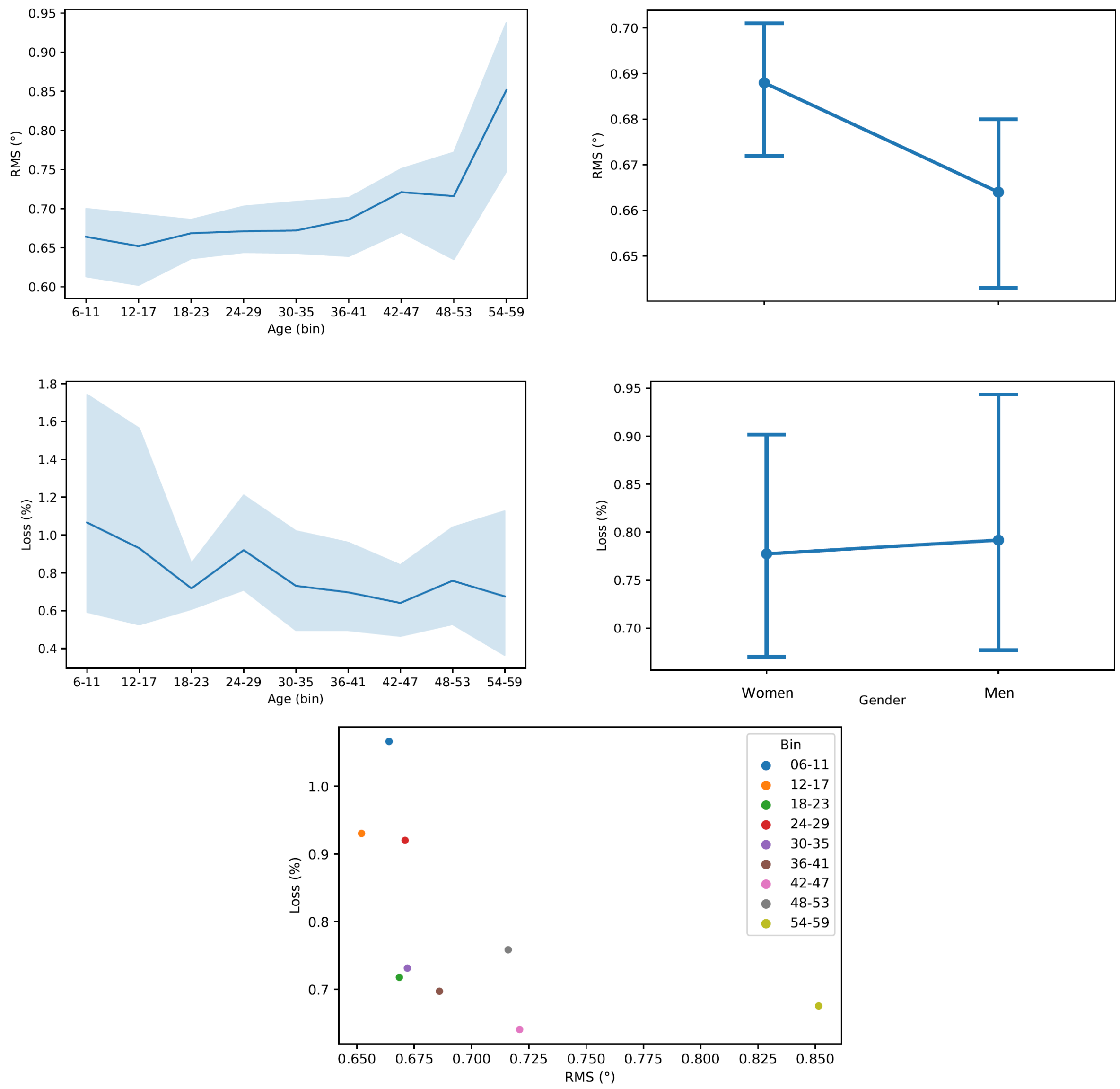

Supplementary Figure 5

Precision across age bins and gender in RMS (upper) and data loss across age bins and gender (middle row). Error bars indicate 95% confidence intervals. Scatter of precision against data loss across age bins (bottom). $n = 1{,}600$.

Graphical summary of individual model predictions

Supplementary Figure 6 depicts spatial distribution maps of actual fixation locations, central bias, and meaning map, as well as all 21 saliency maps evaluated here. Supplementary Figure 7 depicts the difference between spatial distribution maps of actual fixation locations and predicted saliency maps per saliency model.

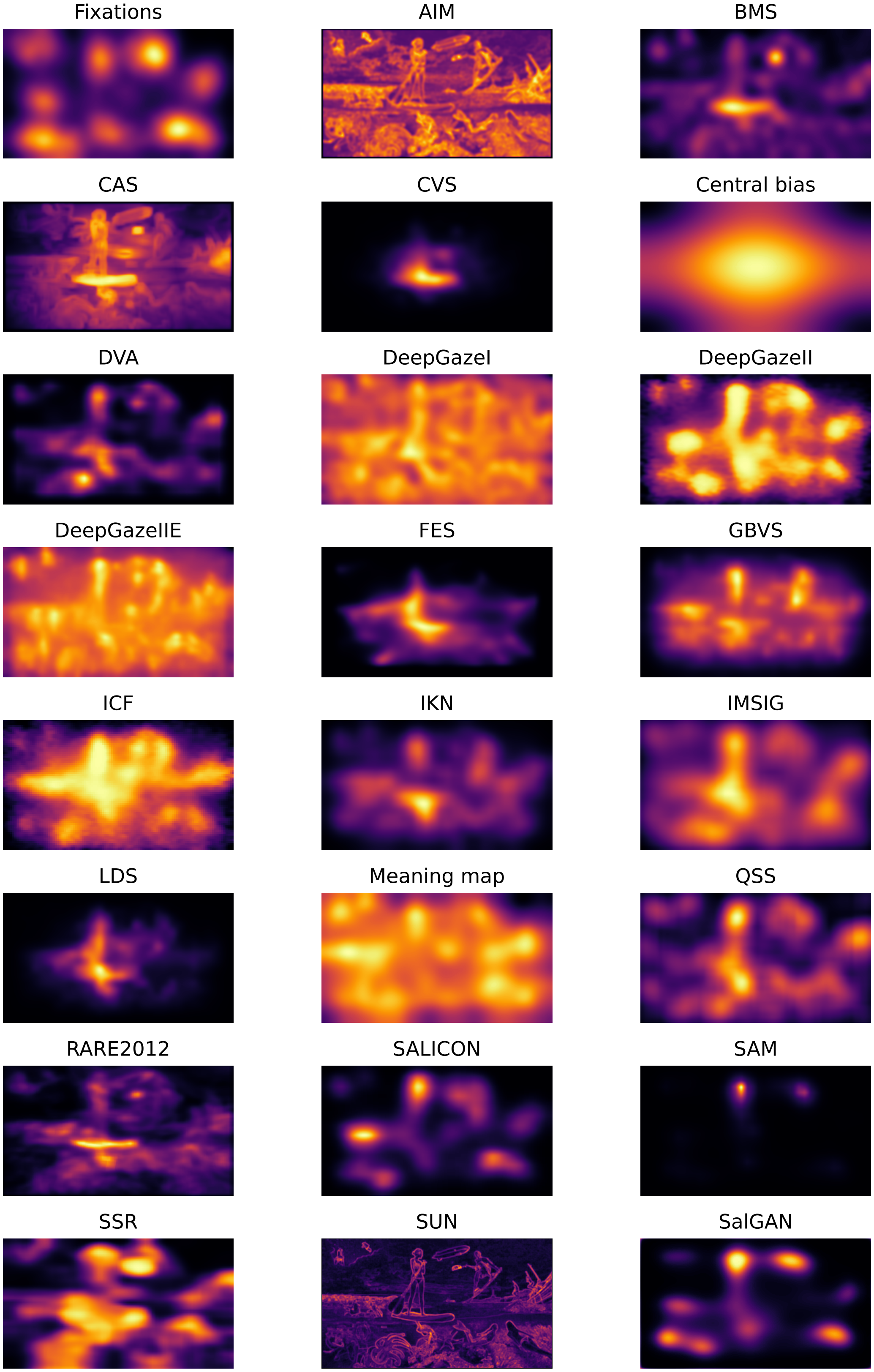

Supplementary Figure 6

All spatial distribution maps. Fixations, central bias, and meaning map are baselines. Other images depict predicted saliency maps per saliency model. Fixation map: $n = 2{,}607$.

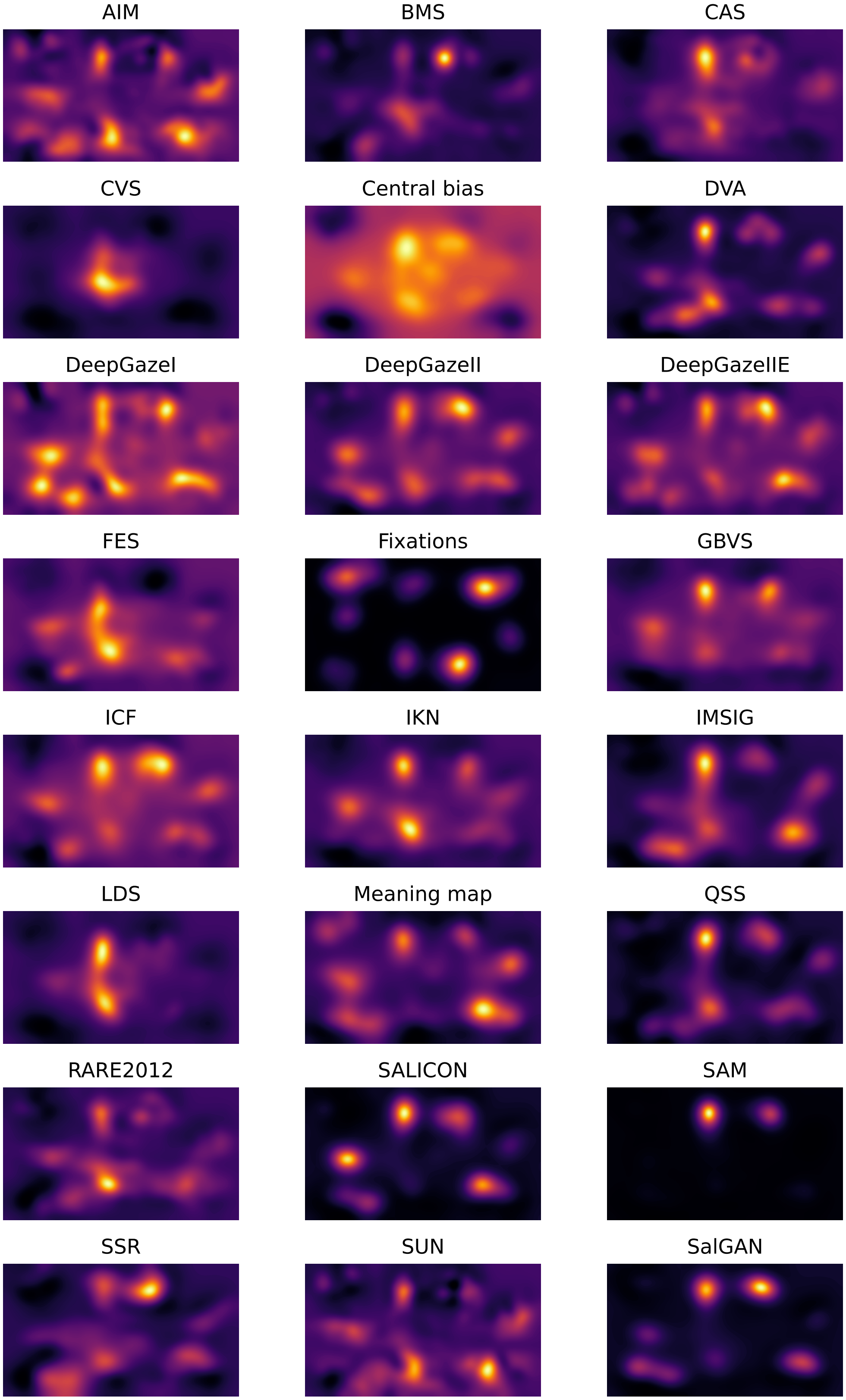

Supplementary Figure 7

Differences between spatial distribution maps of actual fixation locations and predicted saliency per model. 'Fixations' (third row, second column) represents discrete fixation locations against the smoothed fixation map, i.e., the upper bound. n = 2,607.

References

1. Holmqvist, K. *et al.* Eye tracking: empirical foundations for a minimal reporting guideline. *Behavior research methods,* 1–53 (2022).
2. Dunn, M. J. *et al.* Minimal reporting guideline for research involving eye tracking (2023 edition). *Behavior Research Methods* (2023).
3. Hooge, I. T., Niehorster, D. C., Nyström, M., Andersson, R. & Hessels, R. S. Is human classification by experienced untrained observers a gold standard in fixation detection? *Behavior Research Methods* **50,** 1864–1881 (2018).
